# Debunking in a world of tribes

**DOI:** 10.1371/journal.pone.0181821

**Published:** 2017-07-24

**Authors:** Fabiana Zollo, Alessandro Bessi, Michela Del Vicario, Antonio Scala, Guido Caldarelli, Louis Shekhtman, Shlomo Havlin, Walter Quattrociocchi

**Affiliations:** 1 Ca’ Foscari University of Venice, Venezia, Italy; 2 IMT School for Advanced Studies, Lucca, Italy; 3 IUSS, Pavia, Italy; 4 ISC-CNR, Roma, Italy; 5 Bar-Ilan University, Ramat Gan, Israel; Instituto de Fisica Interdisciplinar y Sistemas Complejos, SPAIN

## Abstract

Social media aggregate people around common interests eliciting collective framing of narratives and worldviews. However, in such a disintermediated environment misinformation is pervasive and attempts to *debunk* are often undertaken to contrast this trend. In this work, we examine the effectiveness of debunking on Facebook through a quantitative analysis of 54 million users over a time span of five years (Jan 2010, Dec 2014). In particular, we compare how users usually consuming proven (scientific) and unsubstantiated (conspiracy-like) information on Facebook US interact with specific debunking posts. Our findings confirm the existence of echo chambers where users interact primarily with either conspiracy-like or scientific pages. However, both groups interact similarly with the information within their echo chamber. Then, we measure how users from both echo chambers interacted with 50,220 debunking posts accounting for both users consumption patterns and the sentiment expressed in their comments. Sentiment analysis reveals a dominant negativity in the comments to debunking posts. Furthermore, such posts remain mainly confined to the scientific echo chamber. Only few conspiracy users engage with corrections and their liking and commenting rates on conspiracy posts increases after the interaction.

## Introduction

Socio-technical systems and microblogging platforms such as Facebook and Twitter have created a direct path from producers to consumers of content, changing the way users get informed, debate ideas, and shape their worldviews [1–6]. Misinformation on online social media is pervasive and represents one of the main threats to our society according to the World Economic Forum [7, 8]. The diffusion of false rumors affects public perception of reality as well as the political debate [9]. Indeed, links between vaccines and autism, the belief that 9/11 was an inside job, or the more recent case of Jade Helm 15—a simple military exercise that was perceived as the imminent threat of the civil war in the US—are just few examples of the consistent body of the collective narratives grounded on unsubstantiated information.

Confirmation bias plays a pivotal role in cascades dynamics and facilitates the emergence of echo chambers [10]. Indeed, users online show the tendency a) to select information that adheres to their system of beliefs even when containing parodistic jokes; and b) to join polarized groups [11]. Recently, researches have shown [12–17] that continued exposure to unsubstantiated rumors may be a good proxy to detect gullibility—i.e., jumping the credulity barrier by accepting highly implausible theories—on online social media. Narratives, especially those grounded on conspiracy theories, play an important cognitive and social function in simplifying causation. They are formulated in a way that is able to reduce the complexity of reality and to tolerate a certain level of uncertainty [18–20]. However, conspiracy thinking creates or reflects a climate of disengagement from mainstream society and recommended practices [21].

Several efforts are striving to contrast misinformation spreading from algorithmic-based solutions to tailored communication strategies [22–27] but not much is known about their efficacy. In this work we characterize the consumption of debunking posts on Facebook and, more generally, the reaction of users to dissenting information.

We perform a thorough quantitative analysis of 54 million US Facebook users and study how they consume scientific and conspiracy-like contents. We identify two main categories of pages: conspiracy news—i.e. pages promoting contents *neglected* by main stream media—and science news. Using an approach based on [12, 14, 15], we further explore Facebook pages that are active in debunking conspiracy theses (see section Materials and methods for further details about data collection).

Notice that we do not focus on the quality of the information but rather on the possibility for verification. Indeed, it is easy for scientific news to identify the authors of the study, the university under which the study took place and if the paper underwent a peer review process. On the other hand, conspiracy-like content is difficult to verify because it is inherently based upon suspect information and is derived allegations and a belief in secrets from the public. The self-description of many conspiracy pages on Facebook, indeed, claims that they inform people about topics neglected by mainstream media and science. Pages like *I don’t trust the government*, *Awakening America*, or *Awakened Citizen*, promote wide-ranging content from aliens, chem-trails, to the causal relation between vaccinations and autism or homosexuality. Conversely, science news pages—e.g., *Science*, *Science Daily*, *Nature*—are active in diffusing posts about the most recent scientific advances.

The list of pages has been built by censing all pages with the support of very active debunking groups (see section Materials and methods for more details). The final dataset contains pages reporting on scientific and conspiracy-like news. On a time span of five years (Jan 2010, Dec 2014) we downloaded all public posts (with the related lists of likes and comments) of 83 scientific and 330 conspiracy pages. In addition, we identified 66 Facebook pages aiming at debunking conspiracy theories.

Our analysis shows that two well-formed and highly segregated communities exist around conspiracy and scientific topics—i.e., users are mainly active in only one category. Focusing on users interactions with respect to their preferred content, we find similarities in the consumption of posts. Different kinds of content aggregate polarized groups of users (echo chambers). At this stage we want to test the role of confirmation bias with respect to dissenting (resp., confirmatory) information from the conspiracy (resp., science) echo chamber. Focusing on a set of 50,220 debunking posts we measure the interaction of users from both conspiracy and science echo chambers. We find that such posts remain confined to the scientific echo chamber mainly. Indeed, the majority of likes on debunking posts is left by users polarized towards science (∼67%), while only a small minority (∼7%) by users polarized towards conspiracy. However, independently of the echo chamber, the sentiment expressed by users when commenting on debunking posts is mainly negative.

## Results and discussion

The aim of this work is to test the effectiveness of debunking campaigns on online social media. As a more general aim we want to characterize and compare users attention with respect to a) their preferred narrative and b) information dissenting from such a narrative. Specifically we want to understand how users usually exposed to unverified information such as conspiracy theories respond to debunking attempts.

### Echo chambers

As a first step we characterize how distinct types of information—belonging to the two different narratives—are consumed on Facebook. In particular we focus on users’ actions allowed by Facebook’s interaction paradigm—i.e., likes, shares, and comments. Each action has a particular meaning [28]. A *like* represents a positive feedback to a post; a *share* expresses a desire to increase the visibility of a given information; and a *comment* is the way in which online collective debates take form around the topic of the post. Therefore, comments may contain negative or positive feedbacks with respect to the post.

**Fig 1:**
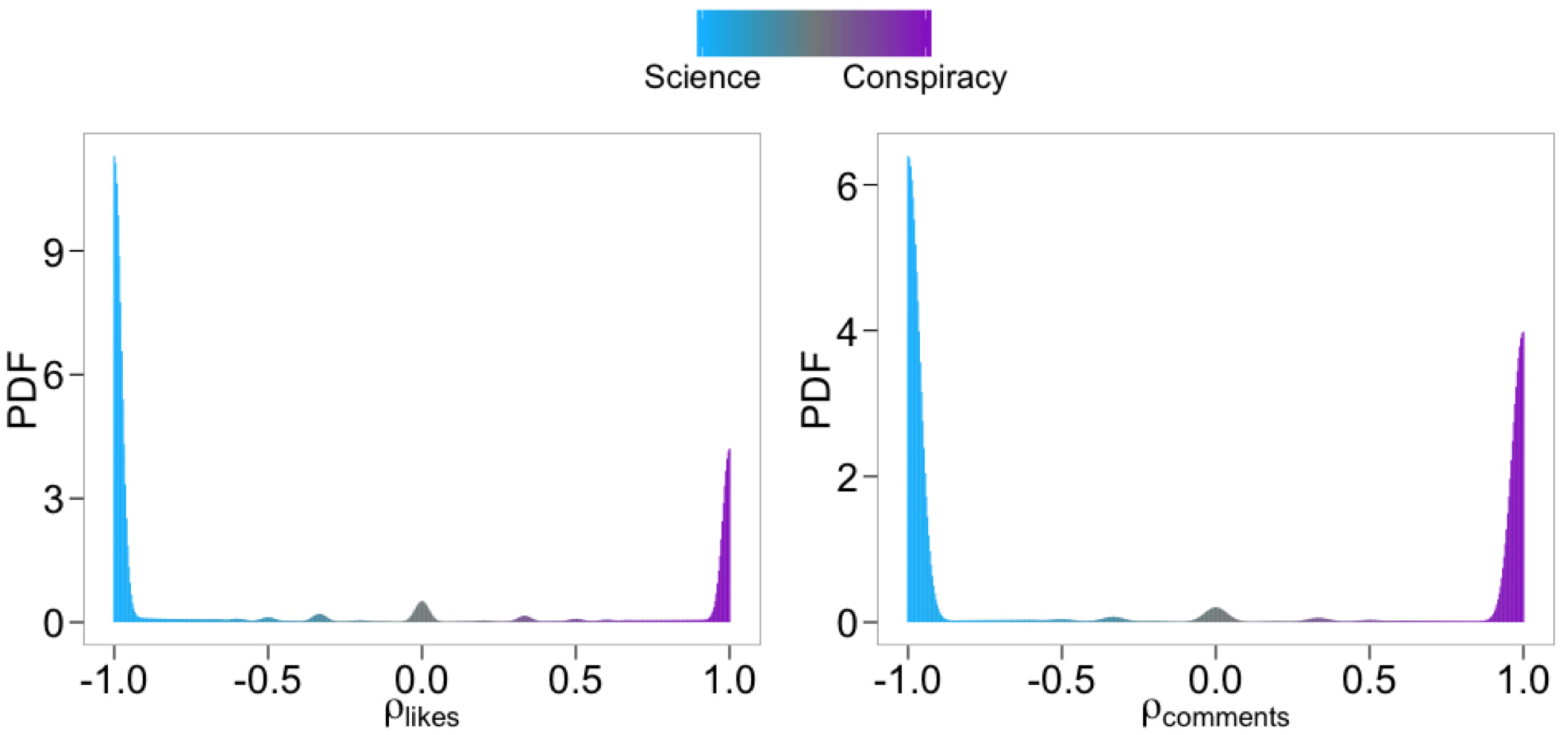
Users polarization. Probability density functions (PDFs) of the polarization of all users computed both on likes *(left)* and comments *(right)*.

Assuming that a user *u* has performed *x* and *y* likes on scientific and conspiracy-like posts, respectively, we let *ρ*(*u*) = (*y* − *x*)/(*y* + *x*). Thus, a user *u* for whom *ρ*(*u*) = −1 is polarized towards science, whereas a user whose *ρ*(*u*) = 1 is polarized towards conspiracy. We define the user polarization *ρ*_*likes*_ ∈ [−1, 1] (resp., *ρ*_*comments*_) as the ratio of difference in likes (resp., comments) on conspiracy and science posts. In Fig 1 we show that the probability density function (PDF) for the polarization of all users is sharply bimodal with most having (*ρ*(*u*) ∼ −1) or (*ρ*(*u*) ∼ 1). Thus, most users may be divided into two groups, those *polarized towards science* and those *polarized towards conspiracy*. The same pattern holds if we look at polarization based on comments rather than on likes.

**Fig 2:**
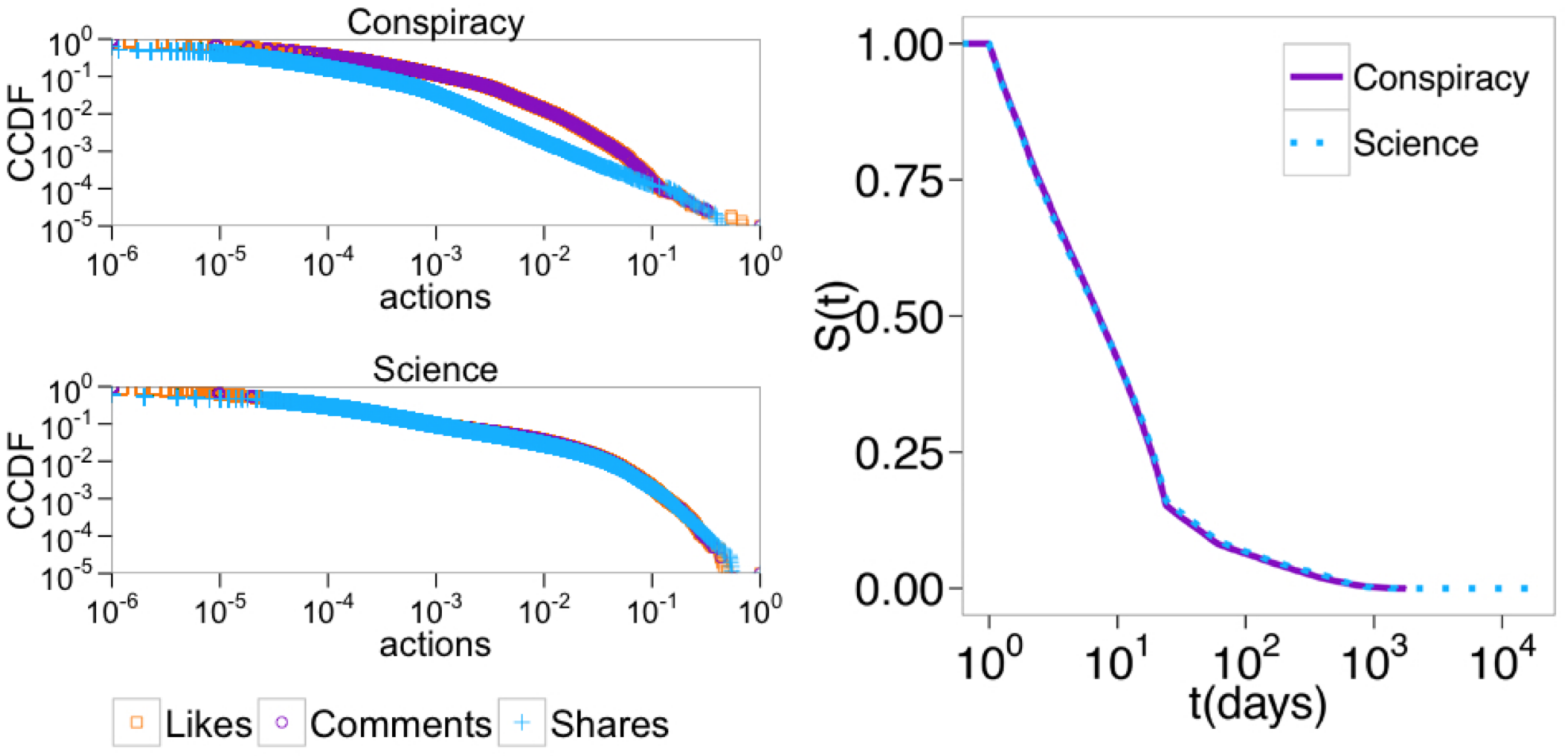
Posts’ attention patterns and persistence. *Left panel:* Complementary cumulative distribution functions (CCDFs) of the number of likes, comments, and shares received by posts belonging to conspiracy *(top)* and scientific *(bottom)* news. *Right panel:* Kaplan-Meier estimates of survival functions of posts belonging to conspiracy and scientific news. Error bars are on the order of the size of the symbols.

To further understand how these two segregated communities behave, we explore how they interact with their preferred type of information. In the left panel of Fig 2 we show the distributions of the number of likes, comments, and shares on posts belonging to both scientific and conspiracy news. As seen from the plots, all the distributions are heavy-tailed—i.e, all the distributions are best fitted by power laws and all possess similar scaling parameters (see Materials and methods section for further details).

We define the persistence of a post (resp., user) as the Kaplan-Meier estimates of survival functions by accounting for the first and last comment to the post (resp., of the user). In the right panel of Fig 2 we plot the Kaplan-Meier estimates of survival functions of posts grouped by category. To further characterize differences between the survival functions, we perform the Peto & Peto [29] test to detect whether there is a statistically significant difference between the two survival functions. Since we obtain a p-value of 0.944, we can state that there are not significant statistical differences between the posts’ survival functions on both science and conspiracy news. Thus, the posts’ persistence is similar in the two echo chambers.

**Fig 3:**
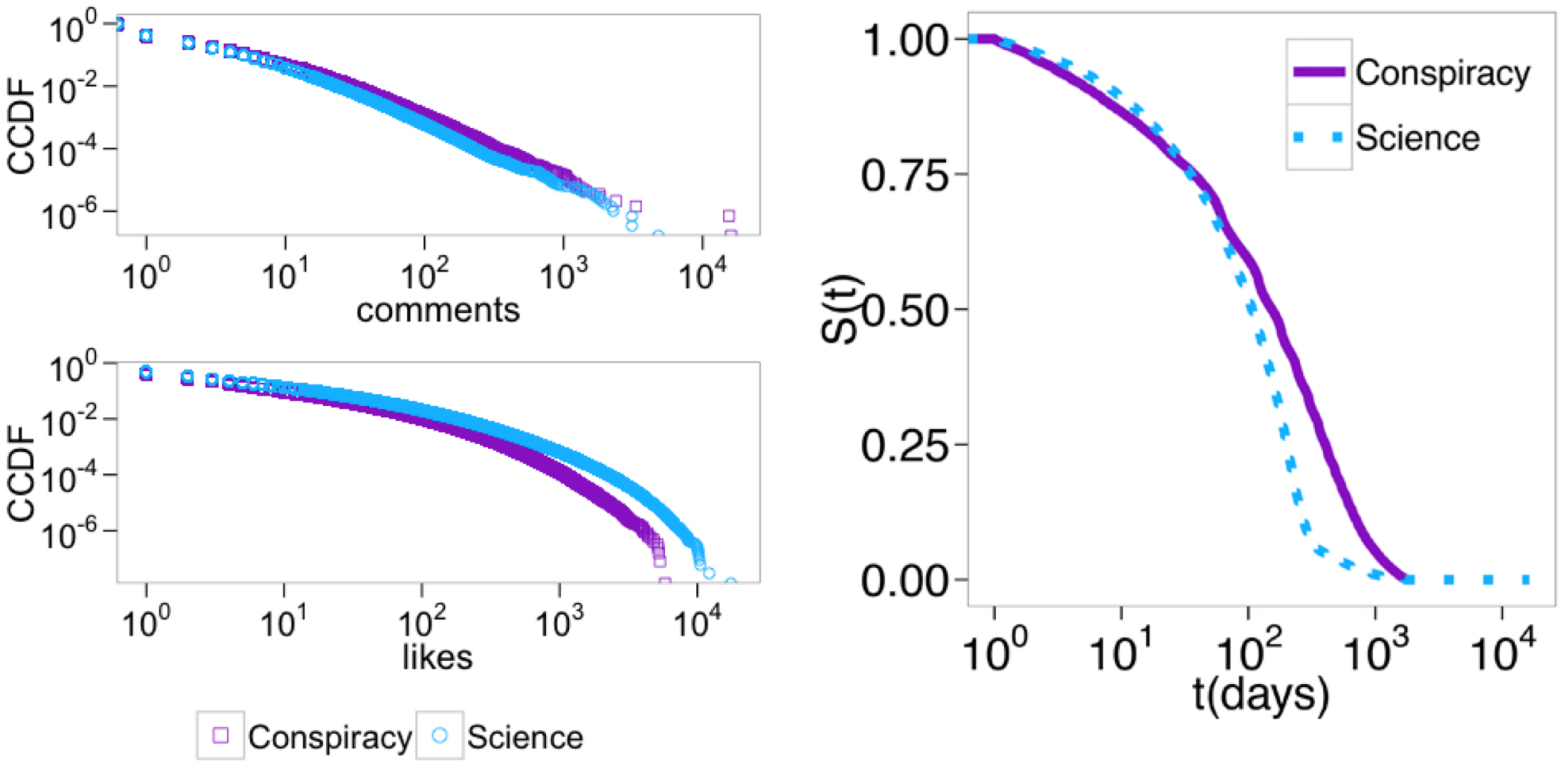
Users’ attention patterns and persistence. *Left panel:* Complementary cumulative distribution functions (CCDFs) of the number of comments *(top)*, and likes *(bottom)*, per each user on the two categories. *Right panel:* Kaplan-Meier estimates of survival functions for users on conspiracy and scientific news. Error bars are on the order of the size of the symbols.

We continue our analysis by examining users interaction with different kinds of posts on Facebook. In the left panel of Fig 3 we plot the CCDFs of the number of likes and comments of users on science or conspiracy news. These results show that users consume information in a comparable way—i.e, all distributions are heavy tailed (for scaling parameters and other details refer to Materials and methods section). The right panel of Fig 3 shows that the persistence of users—i.e., the Kaplan-Meier estimates of survival functions—on both types of content is nearly identical. Attention patterns of users in the conspiracy and science echo chambers reveal that both behave in a very similar manner.

In summary, contents related to distinct narratives aggregate users into different communities and consumption patterns are similar in both communities.

### Response to debunking posts

**Fig 4:**
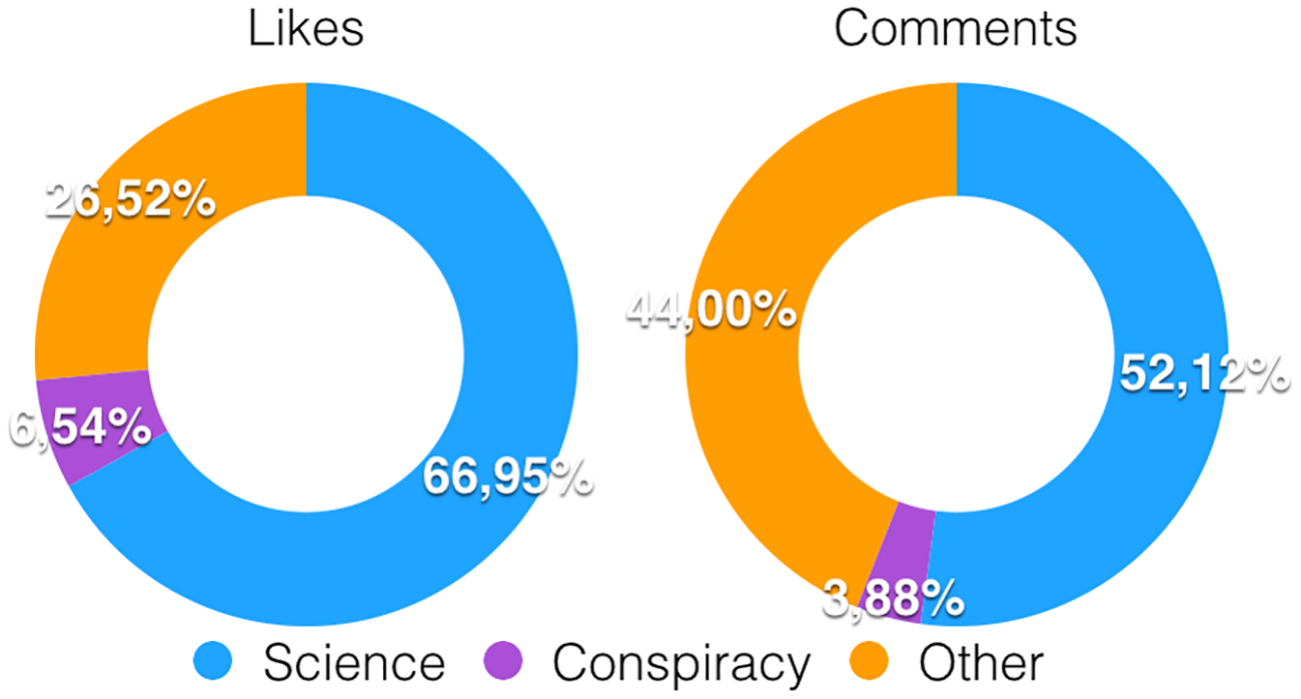
Users’ activity on debunking posts. Proportions of likes *(left)* and comments *(right)* left by users polarized towards science, users polarized towards conspiracy, and not polarized users.

Debunking posts on Facebook strive to contrast misinformation spreading by providing fact-checked information to specific topics. However, not much is known about the effectiveness of debunking to contrast misinformation spreading. In fact, if confirmation bias plays a pivotal role in selection criteria, then debunking might sound to users usually exposed to unsubstantiated rumors like something dissenting from their narrative. Here, we focus on the scientific and conspiracy echo chambers and analyze consumption of debunking posts. As a preliminary step we show how debunking posts get liked and commented according to users polarization. Notice that we consider a user to be polarized if at least the 95% of his liking activity concentrates just on one specific narrative. Fig 4 shows how users’ activity is distributed on debunking posts: Left (resp., right) panel shows the proportions of likes (resp., comments) left by users polarized towards science, users polarized towards conspiracy, and not polarized users. We notice that the majority of both likes and comments is left by users polarized towards science (resp., 66,95% and 52,12%), while only a small minority is made by users polarized towards conspiracy (resp., 6,54% and 3,88%). Indeed, the scientific echo chamber is the biggest consumer of debunking posts and only few users usually active in the conspiracy echo chamber interact with debunking information. Out of 9,790,906 polarized conspiracy users, just 117,736 interacted with debunking posts—i.e., commented a debunking post at least once.

**Fig 5:**
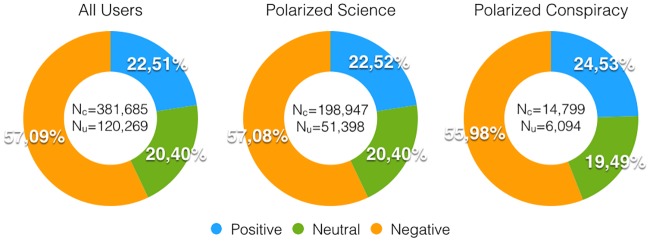
Users’ sentiment on debunking posts. Sentiment of comments made by all users *(left)*, users polarized towards science *(center)*, and users polarized towards conspiracy *(right)* on debunking posts having at least a like, a comment, and a share.

To better characterize users’ response to debunking attempts, we apply sentiment analysis techniques to the comments of the Facebook posts (see Materials and methods section for further details). We use a supervised machine learning approach: first, we annotate a sample of comments and, then, we build a Support Vector Machine (SVM) [30] classification model. Finally, we apply the model to associate each comment with a sentiment value: *negative*, *neutral*, or *positive*. The sentiment denotes the emotional attitude of Facebook users when commenting. In Fig 5 we show the fraction of negative, positive, and neutral comments for all users and for the polarized ones. Notice that we consider only posts having at least a like, a comment, and a share. Comments tend to be mainly negative and such a negativity is dominant regardless of users polarization.

Our findings show that debunking posts remain mainly confined within the scientific echo chamber and only few users usually exposed to unsubstantiated claims actively interact with the corrections. Dissenting information is mainly ignored. Furthermore, if we look at the sentiment expressed by users in their comments, we find a rather negative environment.

#### Interaction with dissenting information

**Fig 6:**
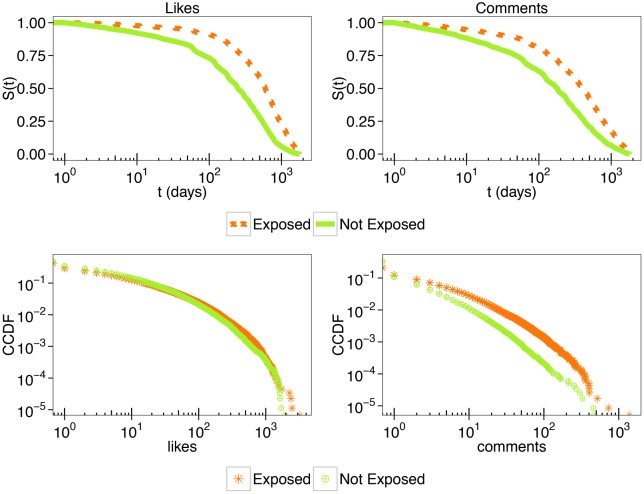
Interaction with debunking: Survival functions and attention patterns. *Top panel:* Kaplan-Meier estimates of survival functions of users who interacted (exposed) and did not (not exposed) with debunking. Users persistence is computed both on their likes *(left)* and comments *(right)*. *Bottom panel:* Complementary cumulative distribution functions (CCDFs) of the number of likes *(left)* and comments *(right)*, per each user exposed and not exposed to debunking.

Users tend to focus on a specific narrative and select information adhering to their system of beliefs while they ignore dissenting information. However, in our scenario few users belonging to the conspiracy echo chamber interact with debunking information. What about such users? And further, what about the effect of their interaction with dissenting information? In this section we aim at better characterizing the consumption patterns of the few users that tend to interact with dissenting information. Focusing on the conspiracy echo chamber, in the top panel of Fig 6 we show the distinct survival functions—i.e. the probability of continuing in liking and commenting along time on conspiracy posts—of users who commented or not on debunking posts. Users interacting with debunking posts are generally more likely to survive—to pursue their interaction with conspiracy posts. The bottom panel of Fig 6 shows the CCDFs of the number of likes and comments for both type of users. The Spearman’s rank correlations coefficient between the number of likes and comments for both type of users are very similar: *ρ*_*exp*_ = 0.53 (95% c.i. [0.529, 0.537]); *ρ*_*not*_*exp*_ = 0.57 (95% c.i. [0.566, 0.573]). However, we may observe that users who commented to debunking posts are slightly *more* prone to comment in general. Thus, users engaging debates with debunking posts seems to be those few who show a higher commenting activity overall.

**Fig 7:**
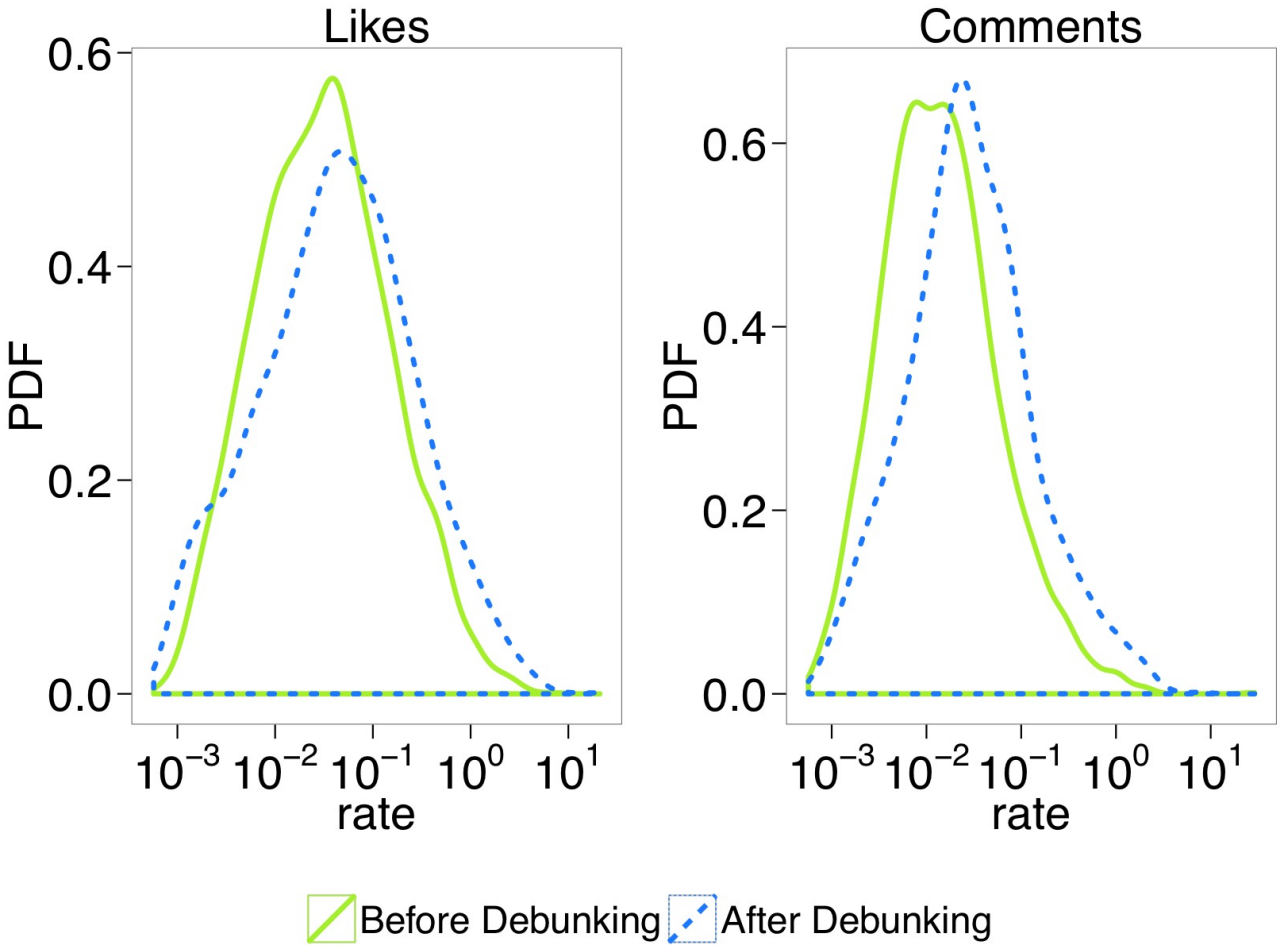
Interaction with debunking: Comments and likes rate. Rate—i.e., average number, over time, of likes *(left)* (resp., comments *(right)*) on conspiracy posts of users who interacted with debunking posts.

To further characterize the effect of the interaction with debunking posts, as a secondary step, we perform a comparative analysis between the users behavior before and after they comment on debunking posts. Fig 7 shows the liking and commenting rate—i.e, the average number of likes (or comments) on conspiracy posts per day—before and after the first interaction with debunking. The plot shows that users’ liking and commenting rates increase after commenting. To assess the difference between the two distributions before and after the interaction with debunking, we perform both Kolmogorov-Smirnov [31] and Mann-Whitney-Wilcoxon [32] tests; since p-value is < 0.01, we reject the null hypothesis of equivalence of the two distributions both for likes and comments rates. To further analyze the effects of interaction with the debunking posts we use the Cox Proportional Hazard model [33] to estimate the hazard of conspiracy users exposed to—i.e., who interacted with—debunking compared to those not exposed and we find that users not exposed to debunking are 1.76 times more likely to stop interacting with conspiracy news (see Materials and methods section for further details).

## Conclusions

Users online tend to focus on specific narratives and select information adhering to their system of beliefs. Such a polarized environment might foster the proliferation of false claims. Indeed, misinformation is pervasive and really difficult to correct. To smooth the proliferation of unsubstantiated rumors major corporations such as Facebook and Google are studying specific solutions. Indeed, examining the effectiveness of online debunking campaigns is crucial for understanding the processes and mechanisms behind misinformation spreading. In this work we show the existence of social echo chambers around different narratives on Facebook in the US. Two well-formed and highly segregated communities exist around conspiracy and scientific topics—i.e., users are mainly active in only one category. Furthermore, by focusing on users interactions with respect to their preferred content, we find similarities in the way in which both forms of content are consumed.

Our findings show that debunking posts remain mainly confined within the scientific echo chamber and only few users usually exposed to unsubstantiated claims actively interact with the corrections. Dissenting information is mainly ignored and, if we look at the sentiment expressed by users in their comments, we find a rather negative environment. Furthermore we show that the few users from the conspiracy echo chamber who interact with the debunking posts manifest a higher tendency to comment, in general. However, if we look at their commenting and liking rate—i.e., the daily number of comments and likes—we find that their activity in the conspiracy echo chamber increases after the interaction.

Thus, dissenting information online is ignored. Indeed, our results suggest that debunking information remains confined within the scientific echo chamber and that very few users of the conspiracy echo chamber interact with debunking posts. Moreover, the interaction seems to lead to an increasing interest in conspiracy-like content.

On our perspective the diffusion of bogus content is someway related to the increasing mistrust of people with respect to institutions, to the increasing level of functional illiteracy—i.e., the inability to understand information correctly—affecting western countries, as well as the combined effect of confirmation bias at work on a enormous basin of information where the quality is poor. According to these settings, current debunking campaigns as well as algorithmic solutions do not seem to be the best options. Our findings suggest that the main problem behind misinformation is conservatism rather than gullibility. Moreover, our results also seem to be consistent with the so-called inoculation theory [34], for which the exposure to repeated, mild attacks can let people become more resistant in changing their ordinary beliefs. Indeed, being repeatedly exposed to relatively weak arguments (*inoculation procedure*) could result in a major resistance to a later persuasive attack, even if the latter is stronger and uses arguments different from the ones presented before i.e., during the inoculation phase. Therefore, when users are faced with untrusted opponents in online discussion, the latter results in a major commitment with respect to their own echo chamber. Thus, a more open and smoother approach, which promotes a culture of humility aiming at demolish walls and barriers between tribes, could represent a first step to contrast misinformation spreading and its persistence online.

## Materials and methods

### Ethics statement

The entire data collection process is performed exclusively by means of the Facebook Graph API [35], which is publicly available and can be used through one’s personal Facebook user account. We used only public available data (users with privacy restrictions are not included in our dataset). Data was downloaded from public Facebook pages that are public entities. Users’ content contributing to such entities is also public unless the users’ privacy settings specify otherwise and in that case it is not available to us. When allowed by users’ privacy specifications, we accessed public personal information. However, in our study we used fully anonymized and aggregated data. We abided by the terms, conditions, and privacy policies of Facebook.

### Data collection

We identified two main categories of pages: conspiracy news—i.e. pages promoting contents *neglected* by main stream media—and science news. Using an approach based on [12, 14], we defined the space of our investigation with the help of Facebook groups very active in debunking conspiracy theses. We categorized pages according to their contents and their self-description. The selection of the sources has been iterated several times and verified by all the authors. To the best of our knowledge, the final dataset is the complete set of all scientific, conspiracist, and debunking information sources active in the US Facebook scenario.

Tables 1–3 show the complete list of conspiracy, science, and debunking pages, respectively. We collected all the posts of such pages over a time span of five years (Jan 2010, Dec 2014). The first category includes all pages diffusing conspiracy information—pages which disseminate controversial information, most often lacking supporting evidence and sometimes contradictory of the official news (i.e. conspiracy theories). Indeed, conspiracy pages on Facebook often claim that their mission is to inform people about topics neglected by main stream media. Pages like *I don’t trust the government*, *Awakening America*, or *Awakened Citizen* promote heterogeneous contents ranging from aliens, chemtrails, geocentrism, up to the causal relation between vaccinations and homosexuality. Notice that we do not focus on the truth value of their information but rather on the possibility to verify their claims. The second category is that of scientific dissemination including scientific institutions and scientific press having the main mission to diffuse scientific knowledge. For example, pages like *Science*, *Science Daily*, and *Nature* are active in diffusing posts about the most recent scientific advances. The third category contains all pages active in debunking false rumors online. We use this latter set as a testbed for the efficacy of debunking campaign. The exact breakdown of the data is presented in Table 4.

**Table 1:** Conspiracy pages.

	Page Name	Facebook ID
1	Spirit Science and Metaphysics	171274739679432
2	Spirit Science	210238862349944
3	The Conspiracy Archives	262849270399655
4	iReleaseEndorphins	297719273575542
5	World of Lucid Dreaming	98584674825
6	The Science of Spirit	345684712212932
7	Esoteric Philosophy	141347145919527
8	9/11 Truth Movement	259930617384687
9	Great Health The Natural Way	177320665694370
10	New World Order News	111156025645268
11	Freedom Isn’t Free on FB	634692139880441
12	Skeptic Society	224391964369022
13	The Spiritualist	197053767098051
14	Anonymous World Wide	494931210527903
15	The Life Beyond Earth	152806824765696
16	Illuminati Exposed	298088266957281
17	Illuminating Souls	38466722555
18	Alternative Way	119695318182956
19	Paranormal Conspiracies	455572884515474
20	CANNABIS CURES CANCERS!	115759665126597
21	Natural Cures Not Medicine	1104995126306864
22	CTA Conspiracy Theorists’ Association	515416211855967
23	Illuminati Killers	478715722175123
24	Conspiracy 2012 & Beyond	116676015097888
25	GMO Dangers	182443691771352
26	The Truthers Awareness	576279865724651
27	Exposing the truth about America	385979414829070
28	Occupy Bilderberg	231170273608124
29	Speak the Revolution	422518854486140
30	I Don’t Trust The Government	380911408658563
31	Sky Watch Map	417198734990619
32	| truthaholics	201546203216539
33	UFO Phenomenon	419069998168962
34	Conspiracy Theories & The Illuminati	117611941738491
35	Lets Change The World	625843777452057
36	Makaveli The Prince Killuminati	827000284010733
37	It’s A New Day	116492031738006
38	New world outlawz—killuminati soldiers	422048874529740
39	The Government’s bullshit. Your argument is invalid.	173884216111509
40	America Awakened	620954014584248
41	The truth behold	466578896732948
42	Alien Ufo And News	334372653327841
43	Anti-Bilderberg Resistance Movement	161284443959494
44	The Truth Unleashed	431558836898020
45	Anti GMO Foods and Fluoride Water	366658260094302
46	STOP Controlling Nature	168168276654316
47	9/11 Blogger	109918092364301
48	9/11 Studies and Outreach Club at ASU	507983502576368
49	9/11 Truth News	120603014657906
50	Abolish the FDA	198124706875206
51	AboveTopSecret.com	141621602544762
52	Activist Post	128407570539436
53	Alliance for Natural Health USA	243777274534
54	All Natural & Organic. Say No To Toxic Chemicals.	323383287739269
55	Alternative Medicine	219403238093061
56	Alternative World News Network	154779684564904
57	AltHealthWORKS	318639724882355
58	American Academy of Environmental Medicine	61115567111
59	American Association of Naturopathic Physicians	14848224715
60	Ancient Alien Theory	147986808591048
61	Ancient Aliens	100140296694563
62	Ancient Astronaut Theory	73808938369
63	The Anti-Media	156720204453023
64	Anti Sodium Fluoride Movement	143932698972116
65	Architects & Engineers for 9/11 Truth	59185411268
66	Association of Accredited Naturopathic Medical Colleges (AANMC)	60708531146
67	Autism Media Channel	129733027101435
68	Babes Against Biotech	327002374043204
69	Bawell Alkaline Water Ionizer Health Benefits	447465781968559
70	CancerTruth	348939748204
71	Chemtrails Awareness	12282631069
72	Collective Evolution	131929868907
73	Conspiracy Theory With Jesse Ventura	122021024620821
74	The Daily Sheeple	114637491995485
75	Dr. Bronner’s Magic Soaps	33699882778
76	Dr. Joseph Mercola	114205065589
77	Dr. Ronald Hoffman	110231295707464
78	Earth. We are one.	149658285050501
79	Educate Inspire Change	467083626712253
80	Energise for Life: The Alkaline Diet Experts!	99263884780
81	Exposing The Truth	175868780941
82	The Farmacy	482134055140366
83	Fluoride Action Network	109230302473419
84	Food Babe	132535093447877
85	Global Research (Centre for Research on Globalization)	200870816591393
86	GMO Inside	478981558808326
87	GMO Just Say No	1390244744536466
88	GreenMedInfo.com	111877548489
89	Healthy Holistic Living	134953239880777
90	I Fucking Love Truth	445723122122920
91	InfoWars	80256732576
92	Institute for Responsible Technology	355853721234
93	I Want To Be 100% Organic	431825520263804
94	Knowledge of Today	307551552600363
95	La Healthy Living	251131238330504
96	March Against Monsanto	566004240084767
97	Millions Against Monsanto by OrganicConsumers.org	289934516904
98	The Mind Unleashed	432632306793920
99	Moms Across America	111116155721597
100	Moms for Clean Air/Stop Jet Aerosol Spraying	1550135768532988
101	Natural Society	191822234195749
102	Non-GMO Project	55972693514
103	Occupy Corporatism	227213404014035
104	The Open Mind	782036978473504
105	Organic Consumers Association	13341879933
106	Organic Health	637019016358534
107	The Organic Prepper	435427356522981
108	PreventDisease.com	199701427498
109	Raw For Beauty	280583218719915
110	REALfarmacy.com	457765807639814
111	ReThink911	581078305246370
112	Sacred Geometry and Ancient Knowledge	363116270489862
113	Stop OC Smart Meters	164620026961366
114	The Top Information Post	505941169465529
115	The Truth About Vaccines	133579170019140
116	Truth Teller	278837732170258
117	Veterans Today	170917822620
118	What Doctors Don’t Tell You	157620297591924
119	Wheat Belly	209766919069873
120	Why don’t you try this?	202719226544269
121	WND	119984188013847
122	WorldTruth.TV	114896831960040
123	Zeitgeist	32985985640
124	Ancient Origins	530869733620642
125	Astrology Answers	413145432131383
126	Astrology News Service	196416677051124
127	Autism Action Network	162315170489749
128	Awakening America	406363186091465
129	Awakening People	204136819599624
130	Cannabinoids Cure Diseases & The Endocannabinoid System Makes It Possible.	322971327723145
131	Celestial Healing Wellness Center	123165847709982
132	Chico Sky Watch	149772398420200
133	A Conscious awakening	539906446080416
134	Conspiracy Syndrome	138267619575029
135	Conspiracy Theory: Truth Hidden in Plain Sight, and Army of SATAN	124113537743088
136	Cosmic Intelligence-Agency	164324963624932
137	C4ST	371347602949295
138	Deepak Chopra	184133190664
139	Dr. Mehmet Oz	35541499994
140	Earth Patriot	373323356902
141	Electromagnetic Radiation Safety	465980443450930
142	EMF Safety Network	199793306742863
143	End Time Headlines	135010313189665
144	Young Living Essential Oils	29796911981
145	Exposing Bilderberg 2012	300498383360728
146	Exposing The Illuminati	196087297165394
147	Exposing Satanic World Government	529736240478567
148	FEMA Camps Exposed	285257418255898
149	Fight Against Illuminati And New World Order	195559810501401
150	FitLife.tv	148518475178805
151	GMO Free USA	402058139834655
152	Holistic Health	105497186147476
153	The Illuminati	543854275628660
154	Illuminati Mind Control	499866223357022
155	Intelwars	130166550361356
156	Natural Solutions Foundation	234136166735798
157	NWO Truth Radio	135090269995781
158	Occupy Bilderberg 2012	227692450670795
159	Operation: Awakening- The Global Revolution	287772794657070
160	The Paradigm Shift	221341527884801
161	PositiveMed	177648308949017
162	Press TV	145097112198751
163	The Resistance	394604877344757
164	Rima E. Laibow, M.D.—Save My Life Dr. Rima	107527312740569
165	RT America	137767151365
166	Ruble’s Wonderings—Forbidden Archeology & Science	265422293590870
167	Seekers Of Truth	736499966368634
168	Spiritual Ecology	261982733906722
169	Spiritualer.com	531950866874307
170	Take Back Your Power	269179579827247
171	There is a cure for Cancer, but it is not FDA approved. Phoenix Tears work!	395190597537
172	True Activist	129370207168068
173	Truth Exposed Radio	173823575962481
174	Truth Movement	161389033958012
175	Truth Network	271701606246002
176	Wake up call	276404442375280
177	We Should Ban GMOs	516524895097781
178	vactruth.com	287991907988
179	Veterans Today Truth Warriors	645478795537771
180	4 Foot Farm Blueprint	1377091479178258
181	Dawning Golden Crystal Age	127815003927694
182	Occupy Your Mind	393849780700637
183	We do not Forgive. We do not Forget. We are Anonymous. Expect Us.	134030470016833
184	Health Impact News	469121526459635
185	NaturalNews.com	35590531315
186	World for 9/11 Truth	38411749990
187	Beware of Disinformation	558882824140805
188	Citizens For Legitimate Government	93486533659
189	Cureyourowncancer.org	535679936458252
190	Juicing Vegetables	172567162798498
191	Quantum Prophecies	323520924404870
192	AIM Integrative Medicine	137141869763519
193	Autism Nutrition Research Center	1508552969368252
194	The Canary Party	220071664686886
195	Chemtrail Research	247681531931261
196	Chemtrail Watchers	77065926441
197	Children’s Medical Safety Research Institute	790296257666848
198	Contaminated Vaccines	686182981422650
199	Dane Wigington	680418385353616
200	David Icke	147823328841
201	David Icke Books Limited	191364871070270
202	David Icke—Headlines	1421025651509652
203	Disinformation Directory	258624097663749
204	The Drs. Wolfson	1428115297409777
205	Educate, Inspire & Change. The Truth Is Out There, Just Open Your Eyes	111415972358133
206	Focus for Health Foundation	456051981200997
207	Generation Rescue	162566388038
208	Geoengineering Watch	448281071877305
209	Global Skywatch	128141750715760
210	The Greater Good	145865008809119
211	The Health Freedom Express	450411098403289
212	Homegrown Health	190048467776279
213	Intellihub	439119036166643
214	The Liberty Beacon	222092971257181
215	International Medical Council on Vaccination	121591387888250
216	International Medical Council on Vaccination—Maine Chapter	149150225097217
217	Medical Jane	156904131109730
218	Mississippi Parents for Vaccine Rights	141170989357307
219	My parents didn’t put me in time-out, they whooped my ass!	275738084532
220	National Vaccine Information Center	143745137930
221	The Raw Feed Live	441287025913792
222	Rinf.com	154434341237962
223	SANEVAX	139881632707155
224	Things pro-vaxers say	770620782980490
225	Unvaccinated America	384030984975351
226	Vaccine Injury Law Project	295977950440133
227	Vermont Coalition for Vaccine Choice	380959335251497
228	9/11: The BIGGEST LIE	129496843915554
229	Agent Orange Activists	644062532320637
230	Age of Autism	183383325034032
231	AutismOne	199957646696501
232	Awakened Citizen	481936318539426
233	Best Chinese Medicines	153901834710826
234	Black Salve	224002417695782
235	Bought Movie	144198595771434
236	Children Of Vietnam Veterans Health Alliance	222449644516926
237	Collective-Evolution Shift	277160669144420
238	Doctors Are Dangerous	292077004229528
239	Dr. Tenpenny on Vaccines	171964245890
240	Dr Wakefield’s work must continue	84956903164
241	EndoRIOT	168746323267370
242	Enenews	126572280756448
243	Expanded Consciousness	372843136091545
244	Exposing the truths of the Illuminati II	157896884221277
245	Family Health Freedom Network	157276081149274
246	Fearless Parent	327609184049041
247	Food Integrity Now	336641393949
248	Four Winds 10	233310423466959
249	Fukushima Explosion What You Do Not Know	1448402432051510
250	The Golden Secrets	250112083847
251	Health Without Medicine & Food Without Chemicals	304937512905083
252	Higher Perspective	488353241197000
253	livingmaxwell	109584749954
254	JFK Truth	1426437510917392
255	New World Order Library | NWO Library	194994541179
256	No Fluoride	117837414684
257	Open Minds Magazine	139382669461984
258	Organic Seed Alliance	111220277149
259	Organic Seed Growers and Trade Association	124679267607065
260	RadChick Radiation Research & Mitigation	260610960640885
261	The REAL Institute—Max Bliss	328240720622120
262	Realities Watch	647751428644641
263	StormCloudsGathering	152920038142341
264	Tenpenny Integrative Medical Centers (TIMC)	144578885593545
265	Vaccine Epidemic	190754844273581
266	VaccineImpact	783513531728629
267	Weston A. Price Foundation	58956225915
268	What On Earth Is Happening	735263086566914
269	The World According to Monsanto	70550557294
270	Truth Theory	175719755481
271	Csglobe	403588786403016
272	Free Energy Truth	192446108025
273	Smart Meter Education Network	630418936987737
274	The Mountain Astrologer magazine	112278112664
275	Alberta Chemtrail Crusaders	1453419071541217
276	Alkaline Us	430099307105773
277	Americas Freedom Fighters	568982666502934
278	Anti-Masonic Party Founded 1828	610426282420191
279	Cannabidiol OIL	241449942632203
280	Cancer Compass An Alternate Route	464410856902927
281	Collective Evolution Lifestyle	1412660665693795
282	Conscious Life News	148270801883880
283	Disclosure Project	112617022158085
284	Dr. Russell Blaylock, MD	123113281055091
285	Dumbing Down People into Sheeple	123846131099156
286	Expand Your Consciousness	351484988331613
287	Fluoride: Poison on Tap	1391282847818928
288	Gaiam TV	182073298490036
289	Gary Null & Associates	141821219197583
290	Genesis II Church of Health & Healing (Official)	115744595234934
291	Genetic Crimes Unit	286464338091839
292	Global Healing Center	49262013645
293	Gluten Free Society	156656676820
294	GMO Free Oregon	352284908147199
295	GMO Journal	113999915313056
296	GMO OMG	525732617477488
297	GreenMedTV	1441106586124552
298	Healing The Symptoms Known As Autism	475607685847989
299	Health Conspiracy Radio	225749987558859
300	Health and Happiness	463582507091863
301	Jesse Ventura	138233432870955
302	Jim Humble	252310611483446
303	Kid Against Chemo	742946279111241
304	Kids Right To Know Club	622586431101931
305	The Master Mineral Solution of the 3rd Millennium	527697750598681
306	Millions Against Monsanto Maui	278949835538988
307	Millions Against Monsanto World Food Day 2011	116087401827626
308	Newsmax Health	139852149523097
309	Non GMO journal	303024523153829
310	Nurses Against ALL Vaccines	751472191586573
311	Oath Keepers	182483688451972
312	Oath Keepers of America	1476304325928788
313	The Organic & Non-GMO Report	98397470347
314	Oregon Coast Holographic Skies Informants	185456364957528
315	Paranormal Research Project	1408287352721685
316	Politically incorrect America	340862132747401
317	(Pure Energy Systems) PES Network, Inc.	183247495049420
318	Save Hawaii from Monsanto	486359274757546
319	Sayer Ji	205672406261058
320	SecretSpaceProgram	126070004103888
321	SPM Southern Patriots MIlitia	284567008366903
322	Thrive	204987926185574
323	Truth Connections	717024228355607
324	Truth Frequency	396012345346
325	Truthstream Media.com	193175867500745
326	VT Right To Know GMOs	259010264170581
327	We Are Change	86518833689
328	Wisdom Tribe 7 Walking in Wisdom.	625899837467523
329	World Association for Vaccine Education	1485654141655627
330	X Tribune	1516605761946273

**Table 2:** Science pages.

	Page Name	Facebook ID
1	AAAS—The American Association for the Advancement of Science	19192438096
2	AAAS Dialogue on Science, Ethics and Religion	183292605082365
3	Armed with Science	228662449288
4	AsapSCIENCE	162558843875154
5	Bridge to Science	185160951530768
6	EurekAlert!	178218971326
7	Food Science	165396023578703
8	Food Science and Nutrition	117931493622
9	I fucking love science	367116489976035
10	LiveScience	30478646760
11	Medical Laboratory Science	122670427760880
12	National Geographic Magazine	72996268335
13	National Science Foundation (NSF)	30037047899
14	Nature	6115848166
15	Nature Education	109424643283
16	Nature Reviews	328116510545096
17	News from Science	100864590107
18	Popular Science	60342206410
19	RealClearScience	122453341144402
20	Science	96191425588
21	Science and Mathematics	149102251852371
22	Science Channel	14391502916
23	Science Friday	10862798402
24	Science News Magazine	35695491869
25	Science-Based Medicine	354768227983392
26	Science-fact	167184886633926
27	Science, Critical Thinking and Skepticism	274760745963769
28	Science: The Magic of Reality	253023781481792
29	ScienceDaily	60510727180
30	ScienceDump	111815475513565
31	ScienceInsider	160971773939586
32	Scientific American magazine	22297920245
33	Scientific Reports	143076299093134
34	Sense About Science	182689751780179
35	Skeptical Science	317015763334
36	The Beauty of Science & Reality.	215021375271374
37	The Flame Challenge	299969013403575
38	The New York Times—Science	105307012882667
39	Wired Science	6607338526
40	All Science, All the Time	247817072005099
41	Life’s Little Mysteries	373856446287
42	Reason Magazine	17548474116
43	Nature News and Comment	139267936143724
44	Astronomy Magazine	108218329601
45	CERN	169005736520113
46	Citizen Science	200725956684695
47	Cosmos	143870639031920
48	Discover Magazine	9045517075
49	Discovery News	107124643386
50	Genetics and Genomics	459858430718215
51	Genetic Research Group	193134710731208
52	Medical Daily	189874081082249
53	MIT Technology Review	17043549797
54	NASA—National Aeronautics and Space Administration	54971236771
55	New Scientist	235877164588
56	Science Babe	492861780850602
57	ScienceBlogs	256321580087
58	Science, History, Exploration	174143646109353
59	Science News for Students	136673493023607
60	The Skeptics Society & Skeptic Magazine	23479859352
61	Compound Interest	1426695400897512
62	Kevin M. Folta	712124122199236
63	Southern Fried Science	411969035092
64	ThatsNonsense.com	107149055980624
65	Science & Reason	159797170698491
66	ScienceAlert	7557552517
67	Discovery	6002238585
68	Critical Thinker Academy	175658485789832
69	Critical Thinking and Logic Courses in US Core Public School Curriculum	171842589538247
70	Cultural Cognition Project	287319338042474
71	Foundation for Critical Thinking	56761578230
72	Immunization Action Coalition	456742707709399
73	James Randi Educational Foundation	340406508527
74	NCSE: The National Center for Science Education	185362080579
75	Neil deGrasse Tyson	7720276612
76	Science, Mother Fucker. Science	228620660672248
77	The Immunization Partnership	218891728752
78	Farm Babe	1491945694421203
79	Phys.org	47849178041
80	Technology Org	218038858333420
81	Biology Fortified, Inc.	179017932138240
82	The Annenberg Public Policy Center of the University of Pennsylvania	123413357705549
83	Best Food Facts	200562936624790

**Table 3:** Debunking pages.

	Page Name	Facebook ID
1	Refutations to Anti-Vaccine Memes	414643305272351
2	Boycott Organic	1415898565330025
3	Contrails and Chemtrails:The truth behind the myth	391450627601206
4	Contrail Science	339553572770902
5	Contrail Science and Facts—Stop the Fear Campaign	344100572354341
6	Debunking Denialism	321539551292979
7	The Farmer’s Daughter	350270581699871
8	GMO Answers	477352609019085
9	The Hawaii Farmer’s Daughter	660617173949316
10	People for factual GMO truths (pro-GMO)	255945427857439
11	The Questionist	415335941857289
12	Scientific skepticism	570668942967053
13	The Skeptic’s Dictionary	195265446870
14	Stop the Anti-Science Movement	1402181230021857
15	The Thinking Person’s Guide to Autism	119870308054305
16	Antiviral	326412844183079
17	Center for Inquiry	5945034772
18	The Committee for Skeptical Inquiry	50659619036
19	Doubtful News	283777734966177
20	Hoax-Slayer	69502133435
21	I fucking hate pseudoscience	163735987107605
22	The Genetic Literacy Project	126936247426054
23	Making Sense of Fluoride	549091551795860
24	Metabunk	178975622126946
25	Point of Inquiry	32152655601
26	Quackwatch	220319368131898
27	Rationalwiki	226614404019306
28	Science-Based Pharmacy	141250142707983
29	Skeptical Inquirer	55675557620
30	Skeptic North	141205274247
31	The Skeptics’ Guide to the Universe	16599501604
32	Society for Science-Based Medicine	552269441534959
33	Things anti-vaxers say	656716804343725
34	This Week in Pseudoscience	485501288225656
35	Violent metaphors	537355189645145
36	wafflesatnoon.com	155026824528163
37	We Love GMOs and Vaccines	1380693538867364
38	California Immunization Coalition	273110136291
39	Exposing PseudoAstronomy	218172464933868
40	CSICOP	157877444419
41	The Panic Virus	102263206510736
42	The Quackometer	331993286821644
43	Phil Plait	251070648641
44	Science For The Open Minded	274363899399265
45	Skeptic’s Toolbox	142131352492158
46	Vaccine Nation	1453445781556645
47	Vaximom	340286212731675
48	Voices for Vaccines	279714615481820
49	Big Organic	652647568145937
50	Chemtrails are NOT real, idiots are.	235745389878867
51	Sluts for Monsanto	326598190839084
52	Stop Homeopathy Plus	182042075247396
53	They Blinded Me with Pseudoscience	791793554212187
54	Pro-Vaccine Shills for Big Pharma, the Illumanati, Reptilians, and the NWO	709431502441281
55	Pilots explain Contrails—and the Chemtrail Hoax	367930929968504
56	The Skeptical Beard	325381847652490
57	The Alliance For Food and Farming	401665083177817
58	Skeptical Raptor	522616064482036
59	Anti-Anti-Vaccine Campaign	334891353257708
60	Informed Citizens Against Vaccination Misinformation	144023769075631
61	Museum of Scientifically Proven Supernatural and Paranormal Phenomena	221030544679341
62	Emergent	375919272559739
63	Green State TV	128813933807183
64	Kavin Senapathy	1488134174787224
65	vactruth.com Exposed	1526700274269631
66	snopes.com	241061082705085

**Table 4:** Breakdown of Facebook dataset. Number of pages, posts, likes, comments, likers, and commenters for science, conspiracy, and debunking pages.

	Total	Science	Conspiracy	Debunking
*Pages*	479	83	330	66
*Posts*	682,455	262,815	369,420	50,220
*Likes*	613,515,345	463,966,540	145,388,131	4,160,674
*Comments*	30,889,614	22,093,692	8,307,643	488,279
*Likers*	52,753,883	40,466,440	19,386,132	744,023
*Commenters*	9,812,332	7,223,473	3,166,725	139,168

### Sentiment classification

Data annotation consists in assigning some predefined labels to each data point. We selected a subset of 24,312 comments from the Facebook dataset (Table 4) and later used it to train a sentiment classifier. We used a user-friendly web and mobile devices annotation platform, Goldfinch—kindly provided by Sowa Labs (http://www.sowalabs.com/)—and engaged trustworthy English speakers, active on Facebook, for the annotations. The annotation task was to label each Facebook comment—isolated from its context—as *negative*, *neutral*, or *positive*. Each annotator had to estimate the emotional attitude of the user when posting a comment to Facebook. During the annotation process, the annotators performance was monitored in terms of the inter-annotator agreement and self-agreement, based on a subset of the comments which were intentionally duplicated. The annotation process resulted in 24,312 sentiment labeled comments, 6,555 of them annotated twice. We evaluate the self- and inter-annotator agreements in terms of Krippendorff’s Alpha-reliability [36], which is a reliability coefficient able to measure the agreement of any number of annotators, often used in literature [37]. *Alpha* is defined as

$$\begin{array}{c} \mathrm{Alpha}=1-\frac{D_{o}}{D_{e}}, \end{array}$$

where *D*_*o*_ is the observed disagreement between annotators and *D*_*e*_ is the disagreement one would expect by chance. When annotators agree perfectly, *Alpha* = 1, and when the level of agreement equals the agreement by chance, *Alpha* = 0. In our case, 4,009 comments were polled twice to two different annotators and are used to assess the inter-annotator agreement, for which *Alpha* = 0.810, while 2,546 comments were polled twice to the same annotator and are used to asses the annotators’ self-agreements, for which *Alpha* = 0.916.

We treat sentiment classification as an ordinal classification task with three ordered classes. We remind that ordinal classification is a form of multi-class classification where there is a natural ordering between the classes, but no meaningful numeric difference between them [38]. We apply the wrapper approach, described in [39], with two linear-kernel Support Vector Machine (SVM) classifiers [30]. SVM is a state-of-the-art supervised learning algorithm, well suited for large scale text categorization tasks, and robust on large feature spaces. The two SVM classifiers were trained to distinguish the extreme classes—*negative* and *positive*—from the rest—*neutral* plus *positive*, and *neutral* plus *negative*. During prediction, if both classifiers agree, they yield the common class, otherwise, if they disagree, the assigned class is *neutral*.

The sentiment classifier was trained and tuned on the training set of 19,450 annotated comments. The comments were processed into the standard Bag-of-Words (BoW) representation. The trained sentiment classifier was then evaluated on a disjoint test set of the remaining 4,862 comments. Three measures were used to evaluate the performance of the sentiment classifier:

1. The aforementioned *Alpha*
2. The *Accuracy*, defined as the fraction of correctly classified examples:
  $$\begin{array}{c} \mathrm{Accuracy}=\frac{\left\langle -,-\rangle +\langle 0,0\rangle +\langle +,+\right\rangle }{N} \end{array}$$
3. $\bar{F_{1}}\left(+,-\right)$, the macro-averaged *F*-score of the positive and negative classes, a standard evaluation measure [40] for sentiment classification tasks:
  $$\begin{array}{c} \bar{F_{1}}\left(+,-\right)=\frac{F_{1}{}_{+}+F_{1}{}_{-}}{2} \end{array}$$
  In general, *F*_1_ is the harmonic mean of *Precision* and *Recall* for each class [41]:
  $$\begin{array}{c} F_{1}=2\cdot \frac{\mathrm{Precision}\cdot \mathrm{Recall}}{\mathrm{Precision}+\mathrm{Recall}} \end{array}$$
  where *Precision* for class *x* is the fraction of correctly predicted examples out of all the predictions with class *x*:
  $$\begin{array}{c} Precision_{x}=\frac{\left\langle x,x\right\rangle }{\left\langle *,x\right\rangle } \end{array}$$
  and *Recall* for class *x* is the fraction of correctly predicted examples out of all the examples with actual class *x*:
  $$\begin{array}{c} Recall_{x}=\frac{\left\langle x,x\right\rangle }{\left\langle x,*\right\rangle } \end{array}$$

The averaged evaluation are the followings: *Alpha* = 0.589±0.017, *Accuracy* = 0.654±0.012, and $\bar{F_{1}}\left(+,-\right)=0.685\;\pm \;0.011$. The 95% confidence intervals are estimated from 10-fold cross validations.

### Statistical tools

#### Kaplan-Meier estimator

Let us define a random variable *T* on the interval [0, ∞), indicating the time an event takes place. The cumulative distribution function (CDF), *F*(*t*) = **Pr**(*T* ≤ *t*), indicates the probability that a subject selected at random will have a survival time less than or equal some stated value *t*. The survival function, defined as the complementary CDF (CCDF), is the probability of observing a survival time greater than some stated value *t*. We remind that the CCDF of a random variable *X* is one minus the CDF, the function *f*(*x*) = **Pr**(*X* > *x*)) of *T*. To estimate this probability we use the *Kaplan–Meier estimator* [42]. Let *n*_*t*_ denote the number of users at risk of stop commenting at time *t*, and let *d*_*t*_ denote the number of users that stop commenting precisely at *t*. Then, the conditional survival probability at time *t* is defined as (*n*_*t*_ − *d*_*t*_)/*n*_*t*_. Thus, if we have *N* observations at times *t*_1_ ≤ *t*_2_ ≤ ⋯ ≤ *t*_*N*_, assuming that the events at times *t*_*i*_ are jointly independent, the Kaplan-Meier estimate of the survival function at time *t* is defined as

$$\begin{array}{c} \hat{S}\left(t\right)=\prod_{t_{i}\leq t}\left(\frac{n_{t_{i}}-d_{t_{i}}}{n_{t_{i}}}\right), \end{array}$$

with the convention that $\hat{S}\left(t\right)=1,\;\text{if}\;\;t<t_{i}$.

#### Comparison between power law distributions

Comparisons between power law distributions of two different quantities are usually carried out through log-likelihood ratio test [43] or Kolmogorov-Smirnov test [31]. The former method relies on the ratio between the likelihood of a model fitted on the pooled quantities and the sum of the likelihoods of the models fitted on the two separate quantities, whereas the latter is based on the comparison between the cumulative distribution functions of the two quantities. However, both the afore-mentioned approaches take into account the overall distributions, whereas more often we are especially interested in the scaling parameter of the distribution, i.e. how the tail of the distribution behaves. Moreover, since the Kolmogorov-Smirnov test was conceived for continuous distributions, its application to discrete data gives biased p-values. For these reasons, in this paper we decide to compare our distributions by assess significant differences in the scaling parameters by means of a Wald test. The Wald test we conceive is defined as

$$\begin{array}{l} H_{0}\;:\;{\hat{\alpha }}_{1}-{\hat{\alpha }}_{2}=0\\ H_{1}\;:\;{\hat{\alpha }}_{1}-{\hat{\alpha }}_{2}\neq 0, \end{array}$$

where ${\hat{\alpha }}_{1}$ and ${\hat{\alpha }}_{2}$ are the estimates of the scaling parameters of the two powerlaw distributions. The Wald statistics,

$$\begin{array}{c} \frac{{\left({\hat{\alpha }}_{1}-{\hat{\alpha }}_{2}\right)}^{2}}{VAR({\hat{\alpha }}_{1})}, \end{array}$$

where $VAR({\hat{\alpha }}_{1})$ is the variance of ${\hat{\alpha }}_{1}$, follows a *χ*^2^ distribution with 1 degree of freedom. We reject the null hypothesis *H*_0_ and conclude that there is a significant difference between the scaling parameters of the two distributions if the p-value of the Wald statistics is below a given significance level.

#### Attention patterns

Different fits for the tail of the distributions have been taken into account (lognormal, Poisson, exponential, and power law). As for attention patterns related to posts, Goodness of fit tests based on the log-likelihood [31] have proved that the tails are best fitted by a power law distribution both for conspiracy and scientific news (see Tables 5 and 6). Log-likelihoods of different attention patterns (likes, comments, shares) are computed under competing distributions. The one with the higher log-likelihood is then the better fit [31]. Log-likelihood ratio tests between power law and the other distributions yield positive ratios, and p-value computed using Vuong’s method [44] are close to zero, indicating that the best fit provided by the power law distribution is not caused by statistical fluctuations. Lower bounds and scaling parameters have been estimated via minimization of Kolmogorov-Smirnov statistics [31]; the latter have been compared via Wald test (see Table 7).

**Table 5:** Goodness of fit for posts’ attention patterns on conspiracy pages.

	Likes	Comments	Shares
*Power law*	**− 34,056.95**	**− 77,904.52**	**− 108,823.2**
*Poisson*	−22,143,084	−6,013,281	−109,045,636
*Lognormal*	−35,112.58	−82,619.08	−113,643.7
*Exponential*	−36,475.47	−87,859.85	−119,161.2

**Table 6:** Goodness of fit for posts’ attention patterns on science pages.

	Likes	Comments	Shares
*Power law*	**− 33,371.53**	**− 2,537.418**	**− 4,994.981**
*Poisson*	−57,731,533	−497,016.2	−3,833,242
*Lognormal*	−34,016.76	−2,620.886	−5,126.515
*Exponential*	−35.330,76	−2,777.548	−5,415.722

**Table 7:** Power law fit of posts’ attention patterns.

	Likes		Comments		Shares	
	${\hat{x}}_{min}$	$\hat{\alpha }$	${\hat{x}}_{min}$	$\hat{\alpha }$	${\hat{x}}_{min}$	$\hat{\alpha }$
*Conspiracy*	8,995	2.73	136	2.33	1,800	2.29
*Science*	62,976	2.78	8,890	3.27	53,958	3.41
*t-stat*	-	0.88	-	325.38	-	469.42
*p-value*	-	0.3477	-	< 10^−6^	-	< 10^−6^

As for users activity, Tables 8 and 9 list the fit parameters with various canonical distributions for both conspiracy and scientific news. Table 10 shows the power law fit parameters and summarizes the estimated lower bounds and scaling parameters for each distribution.

**Table 8:** Goodness of fit for users’ attention patterns on conspiracy pages.

	Likes	Comments
*Power law*	**− 24,044.40**	**− 57,274.31**
*Poisson*	−294,076.1	−334,825.6
*Lognormal*	−25,177.79	−62,415.91
*Exponential*	−28,068.09	−68,650.47

**Table 9:** Goodness of fit for users’ attention patterns on science pages.

	Likes	Comments
*Power law*	**− 222,763.1**	**− 42,901.23**
*Poisson*	−5,027,337	−260,162.7
*Lognormal*	−231,319.1	−46,752.34
*Exponential*	−249,771.4	−51,345.45

**Table 10:** Power law fit of users’ attention patterns.

	Likes		Comments	
	${\hat{x}}_{min}$	$\hat{\alpha }$	${\hat{x}}_{min}$	$\hat{\alpha }$
*Conspiracy*	900	4.07	45	2.93
*Science*	900	3.25	45	3.07
*t-stat*		952.56		17.89
*p-value*		< 10^−6^		2.34×10^−5^

#### Cox-Hazard model

**Fig 8:**
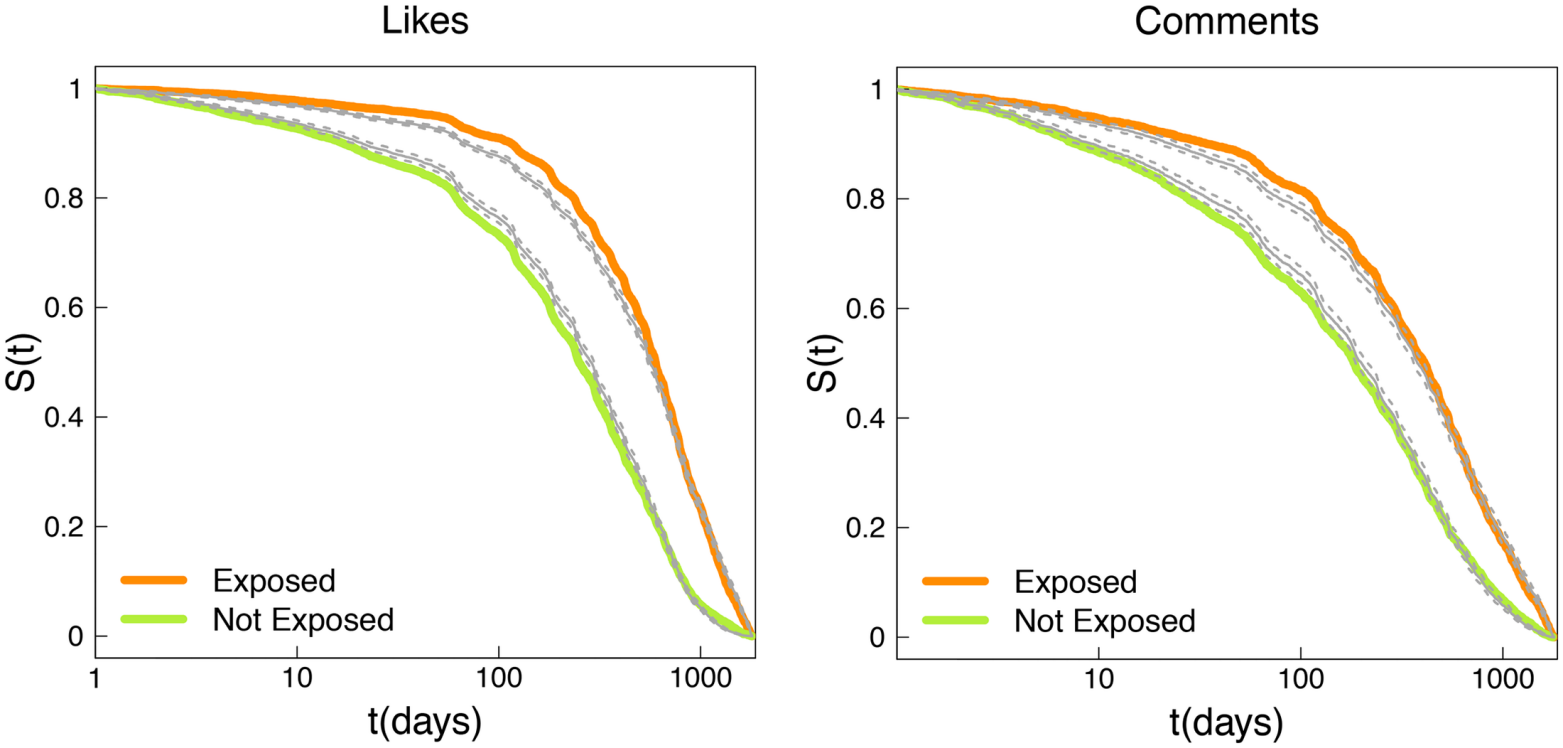
Cox-Hazard model. Kaplan-Meier estimates of survival functions of users who interacted *(exposed, orange)* and did not *(not exposed, green)* with debunking and fits of the Cox proportional hazard model. Persistence of users is computed both on likes *(left)* and comments *(right)*.

The hazard function is modeled as *h*(*t*) = *h*_0_(*t*)exp(*βx*), where *h*_0_(*t*) is the baseline hazard and *x* is a dummy variable that takes value 1 when the user has been exposed to debunking and 0 otherwise. The hazards depend multiplicatively on the covariates, and exp(*β*) is the ratio of the hazards between users exposed and not exposed to debunking. The ratio of the hazards of any two users *i* and *j* is exp(*β*(*x*_*i*_ − *x*_*j*_)), and is called the *hazard ratio*. This ratio is assumed to be constant over time, hence the name of proportional hazard. When we consider exposure to debunking by means of likes, the estimated *β* is 0.72742(*s*.*e*. = 0.01991, *p* < 10^−6^) and the corresponding hazard ratio, exp(*β*), between users exposed and not exposed is 2.07, indicating that users not exposed to debunking are 2.07 times more likely to stop consuming conspiracy news. Goodness of fit for the Cox Proportional Hazard Model has been assessed by means of Likelihood ratio test, Wald test, and Score test which provided p-values close to zero. Fig 8 *(left)* shows the fit of the Cox proportional hazard model when the lifetime is computed on likes.

Moreover, if we consider exposure to debunking by means of comments, the estimated *β* is 0.56748(*s*.*e*. = 0.02711, *p* < 10^−6^) and the corresponding hazard ratio, exp(*β*), between users exposed and not exposed is 1.76, indicating that users not exposed to debunking are 1.76 times more likely to stop consuming conspiracy news. Goodness of fit for the Cox Proportional Hazard Model has been assessed by means of Likelihood ratio test, Wald test, and Score test, which provided p-values close to zero. Fig 8 *(right)* shows the fit of the Cox proportional hazard model when the lifetime is computed on comments.
